# Perceptual response characterization in acute vestibular implant stimulation

**DOI:** 10.1007/s00415-025-13457-7

**Published:** 2025-11-04

**Authors:** B. Volpe, B. L. Vermorken, S. C. J. Van Boxel, N. Guinand, A. Pérez Fornos, E. M. J. Devocht, R. van de Berg

**Affiliations:** 1 Department of Otorhinolaryngology and Head and Neck Surgery, Division of Vestibular Disorders, Maastricht University Medical Center (MUMC+), Maastricht, The Netherlands; 2 Mental Health and Neuroscience Research Institute (MHeNs), Maastricht University, Maastricht, The Netherlands; 3 Division of Otorhinolaryngology Head and Neck Surgery, Department of Clinical Neurosciences, Geneva University Hospitals, Geneva, Switzerland

**Keywords:** Bilateral vestibular loss, Bilateral vestibulopathy, Vestibulocochlear implant, Vestibular implant, Perception, Perceptual response

## Abstract

**Objectives:**

The vestibular implant is a promising treatment option for patients with bilateral vestibulopathy. However, perceptual responses to acute electrical vestibular stimulation remain poorly understood. These perceptual responses are of specific interest as the vestibular system plays a central role in self-motion perception and spatial navigation. This study is the first to systematically examine perceptual responses to acute electrical vestibular stimulation.

**Methods:**

Nine subjects with bilateral vestibulopathy and severe sensorineural hearing loss in the ear to be implanted were included in this study and received an investigational multichannel vestibulocochlear implant. Perceptual responses were assessed for each vestibular electrode across the semicircular canals, over multiple sessions within one year post-implantation. Electrical stimuli were delivered with gradually increasing intensity using a stepwise approach. Following each stimulus, subjects were interviewed about their perceived experiences using an open, semi-structured approach. Responses were categorized by perception type, and thresholds were analyzed relative to stimulation intensity and the targeted ampullary nerve.

**Results:**

Three main types of perceptual responses were identified: motion, auditory, and vibration. Motion perception roughly aligned with the axis of the stimulated canal. Auditory perceptions increased in pitch with increasing stimulation amplitude. Vibration was consistently reported across all subjects and vestibular electrodes. Perceived intensity increased linearly with stimulation amplitude, from low threshold to upper comfortable level.

**Conclusion:**

This study structurally characterized perceptual responses to electrical vestibular stimulation in subjects with a vestibulocochlear implant for the first time. The identification of distinct response types and their relationship to stimulus parameters provides a foundation for improving implant fitting and optimizing stimulation paradigms. Future studies should refine fitting strategies based on these perceptual findings.

## Introduction

Bilateral vestibulopathy, defined as severe bilateral vestibular function loss, is an often missed or misdiagnosed disorder [1]. It is mainly characterized by chronic symptoms of oscillopsia and unsteadiness, which worsen in darkness or when on uneven terrain [2–5]. These symptoms often lead to physical, cognitive and emotional complaints, resulting in a detrimental effect on the subjects’ quality of life [6–8]. Current treatment options are limited and mainly focus on physical rehabilitation. Unfortunately, the effects of rehabilitation remain poor, with more than 80% of subjects not improving [9–11].

Much effort has been put into finding new treatment options for this patient population. One such effort is the development of the vestibular implant (VI). The VI is a device capable of capturing head motion information and transferring this information directly to the vestibular nerves using implanted electrodes. This is analogous to the way a cochlear implant (CI) transfers sound information to the cochlear nerve in patients with severe sensorineural hearing loss (SNHL). The Geneva-Maastricht Group aims to restore both vestibular and hearing function in subjects with BV and severe SNHL, by using a vestibulocochlear implant (VCI). The VCI combines three VI branches, targeting the three semicircular canals, with a CI array targeting the cochlea. Previous research, by multiple research groups around the world, has explored stimulating either the semicircular canals or the otolith organs and already showed feasibility of the VI concept [12–17].

Most research on the VI focuses on objective outcome measures, particularly the restoration of the vestibulo-ocular reflex (VOR) [15, 18–22] or vestibulo-collic reflex [23]. More subjective outcomes, like perceptual responses to electrical vestibular stimulation, are often briefly mentioned but not yet reported in detail. These perceptual responses to electrical vestibular stimulation are important to study, because the vestibular system plays a central role in self-motion perception and spatial navigation. The vestibular system comprises five specialized receptors: three semicircular canals and two otolith organs (utricle and saccule). The semicircular canals encode information about angular head motion, whereas the otoliths encode information about linear acceleration and gravity. Information from these receptors is transmitted via the vestibulocochlear nerve to the central vestibular pathways. This information is then further processed at multiple levels, including the vestibular nuclei, cerebellum, thalamus and cortex. At these levels, the vestibular information is integrated with visual, proprioceptive and motor signals, contributing significantly to our perception of self-motion and ability to navigate. Because of this integration with other sensory signals, there is no distinct or conscious sensation arising from the vestibular system in everyday life [24–27].

In patients with BV, this perception of self-motion is known to be distorted, leading to higher self-motion detection thresholds [24, 28–33]. Additionally, studies using noisy galvanic stimulation demonstrated that electrical stimulation of the vestibular system can alter and potentially improve vestibular perception. This was observed in both healthy subjects [34–37] and BV patients [38].

Previously, different types of sensations in response to vestibular stimulation by a VI were reported by the Geneva–Maastricht group [17, 39]. Briefly, sound was the most frequently reported perception when stimulating the posterior ampullary nerve (PAN). Stimulating the lateral and superior ampullary nerves (LAN and SAN, respectively) evoked a wider range of sensations, including rotational movement, sound, tickling, and pressure. These perceptions were only raw descriptions of the first sensations reported at the lowest stimulation intensity, in a limited number of subjects and at only one test session. However, these initial results did not yet provide insight into perceptions across the full range of stimulable intensities. A research group at Johns Hopkins, also using a semicircular canal VI, reported vertigo and motion sensations after activation of the VI [15]. However, these perceptions were not further specified. In another study, a research group at the University of Washington reported more extensively on perception in subjects with Meniere’s disease [40]. It was reported that electrical stimulation of LAN, SAN, and PAN produced different perceptual responses. For example, LAN stimulation induced yaw rotation, SAN stimulation caused sensations of being pushed forward and down, and PAN stimulation elicited backward pitch rotation. Two subjects also experienced a sensation of high-frequency vibration. Activation of an otolith implant, on the other hand, resulted in an initial feeling of surprise, followed by sensations of comfort and stability as reported by two subjects while seated [14]. When the intensity of the stimulation increased excessively, they experienced unexpected motion sickness without accompanying nausea.

These findings underscore the importance of understanding how subjects perceive direct electrical vestibular stimulation. Such insights could help in further optimizing VI fitting in the future, potentially restoring functions like self-motion perception. Moreover, the role of vestibular perception in VI fitting was already demonstrated by its use in determining the electrical dynamic range of the vestibular implant [14, 15, 17, 41]. Typically, the first (vestibular) perception is used to establish the threshold of the dynamic range, whereas the upper comfortable limit (UCL) is determined by the maximum tolerated current. It was previously suggested that incorporating perceptual responses in determining stimulation parameters and transfer functions may significantly change the VI fitting procedure [42].

To our knowledge, this study was the first to systematically and comprehensively examine perceptual responses to acute electrical vestibular stimulation. More specifically, it focused on assessing the different types of perceptual responses, their thresholds, and their intensities in relation to stimulation intensity and the stimulated semicircular canals.

## Methods

### Overall study design

Nine subjects with BV and severe sensorineural hearing loss in the ear to be implanted were included in this study. After surgical implantation of a VCI, the VCI was fitted and perceptual responses of every vestibular electrode were investigated at least five times over the course of one year. During each test session, electrodes were stimulated separately using block stimuli of two seconds. These stimuli were increased in amplitude using a stepwise approach. After each given stimulation, the subjects were asked to report their perception. Questioning included several aspects: 1) type of perception (e.g., sound, motion), 2) quality of the perception (e.g., low- or high-pitched sound, horizontal or vertical movement), and 3) intensity of the perception.

### Subjects and surgical implantation

All nine subjects participated in the VertiGO! trial and fulfilled the inclusion criteria, based on the vestibular implantation criteria opinion statement [43]. A full list of in- and exclusion criteria, as well as the whole study protocol, was previously described in detail [44]. Subject characteristics are presented in Table 1.

**Table 1:** Subject characteristics

Subject ID	Sex	Age at implantation (years)	Etiology	Duration of BV symptoms (years)	Implanted side	Year of implantation
VCI-01	F	54	DFNA-9	7	Right	2021
VCI-02	M	65	Auto-immune (CREST)	21	Right	2021
VCI-03	M	52	DFNA-9	30	Left	2022
VCI-04	M	66	DFNA-9	10	Right	2022
VCI-05	M	28	Idiopathic	4	Right	2022
VCI-06	M	66	Menière’s Disease	25	Right	2022
VCI-07	F	62	DFNA-9	6	Left	2022
VCI-08	M	63	Skull base fracture	< 1	Right	2023
VCI-09	F	62	Skull base fracture	< 1	Right	2023

All subjects were implanted with an investigational vestibulocochlear implant (supplied to the clinic by MED-EL, Innsbruck, Austria) between 2021 and 2023. This device, a modified cochlear implant, has three vestibular electrodes and one cochlear lead with nine electrodes. The vestibular electrodes were inserted in each of the three semicircular canals and positioned within the ampullae. The cochlear lead was inserted into the cochlea. VI implantation was performed using the intralabyrinthine approach in all cases, as previously described [44–46]. Postoperatively, subjects received standard clinical CI rehabilitation.

### Study visits

As part of the VertiGO! trial, for each patient, multiple fitting sessions were scheduled over the course of one year postoperatively. Perceptual responses across vestibular electrodes were investigated at each of these sessions. The first test sessions were scheduled at approximately 6, 11, and 16 weeks postoperatively. Once the first three months of intensive CI rehabilitation were completed, four days of VI fitting were scheduled, during which each vestibular electrode was investigated twice.

### Experimental setup

#### Electrical stimulation

During electrical stimulation, subjects sat upright in a fixed chair. Video-oculography (VOG) goggles (Interacoustics, Middelfart, Denmark) prevented visual fixation. The lights in the room were dimmed.

To deliver electrical stimulation, a radiofrequency coil was positioned on the subcutaneous implant receiver. The stimulation was controlled using a laptop running either the MAESTRO™ software or custom AMPFit™ research software, both supplied by MED-EL (Innsbruck, Austria). Communication between the laptop and the radiofrequency coil was achieved using the MAX interface box (MED-EL, Innsbruck, Austria). Each stimulus consisted of a 2-s pulse train of rectangular, charge-balanced, cathodic-first, biphasic pulses (200 µs/phase) presented at the maximum pulse rate (322–400 pulses/s), depending on the configuration of the patient’s individual CI. These pulses were delivered to each vestibular electrode separately. Cochlear electrodes were deactivated during stimulation to prevent any potential interference.

First, a vestibular threshold was determined for each vestibular electrode. The threshold was defined as the lowest current (in current units) at which one or more of the following two criteria were met: (1) perception(s) reported by the subject, and/or (2) VOR response observed by the examiners. The initial stimulation amplitude was set at 50 cu and was incremented by 50 cu steps until the vestibular threshold was reached. The vestibular threshold was then confirmed by lowering the intensity by 25 cu to ensure that none of the threshold criteria were met at the lower intensity level, followed by re-evaluating the initial intensity level at which the vestibular threshold was identified.

After vestibular threshold determination, stimulation intensity was further increased in steps of 50 cu until the upper comfortable limit (UCL) was reached. The UCL was defined as the current (cu) just below the point at which one or more of the following three criteria were met: (1) the subject reported an uncomfortable response, scored as maximum on a custom-made visual-analog scale (VAS, see below), and/or (2) evident stimulation of the facial nerve was present, and/or (3) the maximum current level that could be delivered by the device was reached. The electrical dynamic range was then defined as the current range between the vestibular threshold and UCL.

#### Perceptual response recording

Perceptual responses were collected after each stimulation across the dynamic range (see above). After each stimulation, the lid of the VOG goggles was removed to facilitate communication. Subjects were interviewed regarding their perceptions using an open, semi-structured approach. This allowed the subjects to describe their perceptions without bias or assumptions.

Subjects were first asked if they felt any sensations (1) type of perception. If they did, they were encouraged to elaborate and describe the nature of their sensations (2) quality of perception. If a motion percept was reported, subjects were asked to specify its direction. The intensity of the perceptions was noted using a custom-made VAS (3) intensity of perception. The VAS ranged from 0 to 10, where 0 indicated no percept and 10 indicated a sensation that was too strong or too uncomfortable. Afterward, subjects were specifically asked about any auditory perceptions during the stimulation.

### Perceptual response data processing

#### Categorization of perceptual responses

Subjects reported numerous perceptual responses during electrical vestibular stimulation. All responses were noted down in keywords after being reported by the subjects. To facilitate analysis, responses were categorized and classified through a systematic process. The classification process consisted of three key steps:

First, an initial screening of the reported perceptions was conducted by the first author (BV). Based on this screening, it was found by consensus (BV, EDV, and RVDB) that three main types of perceptual responses were present: motion perception, auditory perception, and vibration perception. These perception types formed the basis to further explore the specific qualities of perceptual responses.

In addition to motion, auditory, and vibration sensations, a small number of reports described non-canonical descriptors such as general ‘dizziness’ or facial-nerve–like sensations ‘tingling/twitching’. These descriptors were reported infrequently and inconsistently (e.g., dizziness was reported intermittently by 2 out of 9 subjects and did not occur across all sessions). Notably, all non-canonical descriptors were co-reported with motion or vibration sensations and were therefore treated as modifiers rather than as a distinct class. Nevertheless, their verbatim descriptions were retained in the dataset.

Next, subcategories were created for each perception type, based on the initial screening (see Table 2). For motion perception, quality subcategories were defined as related to the direction of movement. When participants reported a within-trial transition of direction, the terminal (most salient) direction was used for categorical analyses; ambiguous transitions were coded as ‘Oblique’, and the verbatim description was retained. For auditory perception, quality subcategories were identified as related to the type of sound. The first author (BV) then classified all perceptual data according to these quality subcategories. If there was any uncertainty regarding the categorization of a subject’s response, authors BV, EDV, and RVDB reached a consensus on the final classification.

**Table 2:** Perceptual response categorization of electrical vestibular stimulation. Responses were classified by Perception Type (motion, auditory, and vibration), further refined into Perception Quality—Main Category (such as direction for movement, or pitch for auditory perception), and detailed by Perception Quality—Subcategory

Perception type	Perception quality – main category	Perception quality – subcategory
Motion	Horizontal	Horizontal, Horizontal left, Horizontal right
	Vertical	Vertical, Vertical upward, Vertical downward
	Oblique	Oblique, Oblique left, Oblique right, Oblique upward, Oblique downward, Oblique left-upward, Oblique left-downward, Oblique right-downward, oblique right-upward
	None	None
	Unknown	Unknown
Auditory	Low pitch	Low zoom, hum
	High pitch	High zoom, beep, whistle, screeching
	None	None
	Unknown	Unknown
Vibration	Vibration	Vibration, current, buzz, wave, hum, pulse
	None	None

In some cases, not all perceptions could be precisely categorized according to quality into the defined subcategories. To address this, the subcategories were summarized into broader umbrella categories (‘main categories’, see Table 2). This summarization was necessary because subjects’ responses regarding the quality of the percept could not always be assigned to a specific subcategory but could be classified in a broader main category. For example, a subject might report a horizontal perception (main category) but was unable to specify whether it involved movement to the left or right (subcategory). The same principle applied to auditory perception. Vibration perception was categorized as a single type. The resulting main categories were used for the final analysis of the data.

#### Extrapolation

Due to the extensive number of stimulations and associated perceptual responses collected during prolonged, attention-intensive sessions, some degree of data loss was unavoidable. To ensure methodological consistency and maintain data integrity, a structured extrapolation procedure was employed to address instances of missing or incomplete perceptual responses. Missingness occurred primarily due to participants’ inattention during testing or recording errors. In such cases, surrounding data were used to infer the most plausible response. Extrapolation was applied in the following manner in the described scenarios: (a) if no perception was recorded for a certain stimulation intensity, but the previous and subsequent intensities showed the same perception, extrapolation was applied for the unrecorded stimulation intensity, and/or (b) when a particular perception was observed at a specific stimulation intensity, not observed for a few subsequent levels, and then reappeared, it was assumed that the perception was also present at the intervening levels, and/or (c) if a certain stimulation intensity was repeated and no perception was recorded, but the perception was noted in previous repetitions at that intensity, the previous perception was extrapolated, and/or (d) if a sound was perceived at a certain level but not at higher levels, based on prior experience of the researchers, it was assumed that the sound was also present at the higher stimulation levels.

### Data analysis and statistics

All analyses were carried out in R (v.4.3.3) and IBM SPSS Statistics (v. 28.0, IBM Corp, Armonk NY, USA), with a focus on descriptive statistics. Visual representations were used to identify patterns and trends in the data. Analysis was structured to address the three goals (type of perception, quality of perception, and intensity of perception):

First, a general overview of all occurring perception types (motion, auditory, vibration) was created. The proportion of each perception type was calculated relative to the total number of stimulation presentations. Data were grouped by subject and electrode and then classified as either present or absent. A perception type was considered significantly present if it occurred in > 10% of stimulations within the dynamic range. If a certain perception type appeared in ≤ 10% of the stimulations, it was concluded that this perception was not significantly present. Additionally, the individual and group average perception thresholds were calculated for each perception type. The threshold of the perception type was defined as the lowest stimulation intensity at which a subject reported the perception type. The stimulation intensity was normalized for each subject and electrode during each study visit by expressing it as a percentage of the dynamic range. The threshold was set at 0%, and the UCL at 100%. This normalization enabled the comparison of perceptions between subjects and across study visits.

Second, perception quality was analyzed per perception type and vestibular electrode to evaluate how perceptual characteristics varied across the dynamic range. Because it was believed that the different perception qualities of vibration all referred to the same perception, vibration perception was investigated in relation to motion perception. As some stimulations exceeded the UCL (as part of its determination), a stimulation intensity range of 0–125% was used for analysis. To improve visual representation, the stimulation intensity range was divided into 25% bins. Only data from subjects in which a perception type was significantly present (> 10%) were included in the corresponding analysis of the perception type. Consequently, data from subjects VCI-03 and VCI-04 were excluded from motion perception analysis, and data from subjects VCI-01, VCI-02, and VCI-06 were excluded from auditory perception analysis. To ensure equal representation of each subject, weights were assigned to each measurement. First, the data were grouped by electrode, dynamic range bin, and subject. Then, the number of stimulations within each group was counted. The weight applied to each measurement was equal to the inverse of the number of measurements within each group. These weights were then used for further analysis. This weighting approach balanced the contribution of each subject in every bin of the dynamic range, preventing overrepresentation by those with a higher number of measurements (i.e., when a subject has a larger dynamic range).

Third, perception intensity was assessed by plotting the VAS scores against the stimulation intensity (as a percentage of dynamic range) per electrode. A mixed linear regression model, incorporating a random intercept for subject and study visit, and a random slope for stimulation intensity per subject, was used to investigate the predictive relationship between perception intensity (VAS score) and the dynamic range per electrode. P-values were computed using a Wald t-distribution approximation.

## Results

A total of 1367 perceptions were recorded from nine subjects. Stimulation intensity ranged from 25 to 950 cu across subjects. Of these 1367 data points, 35 (2.6%) were explicitly missing and extrapolated. In addition, a number of incomplete perceptual records were complemented at the response level, ensuring that the dataset was largely complete and internally consistent. Initial screening of the perceptual response data identified three distinct types of perceptual responses: motion, auditory, and vibration percept.

**Fig. 1:**
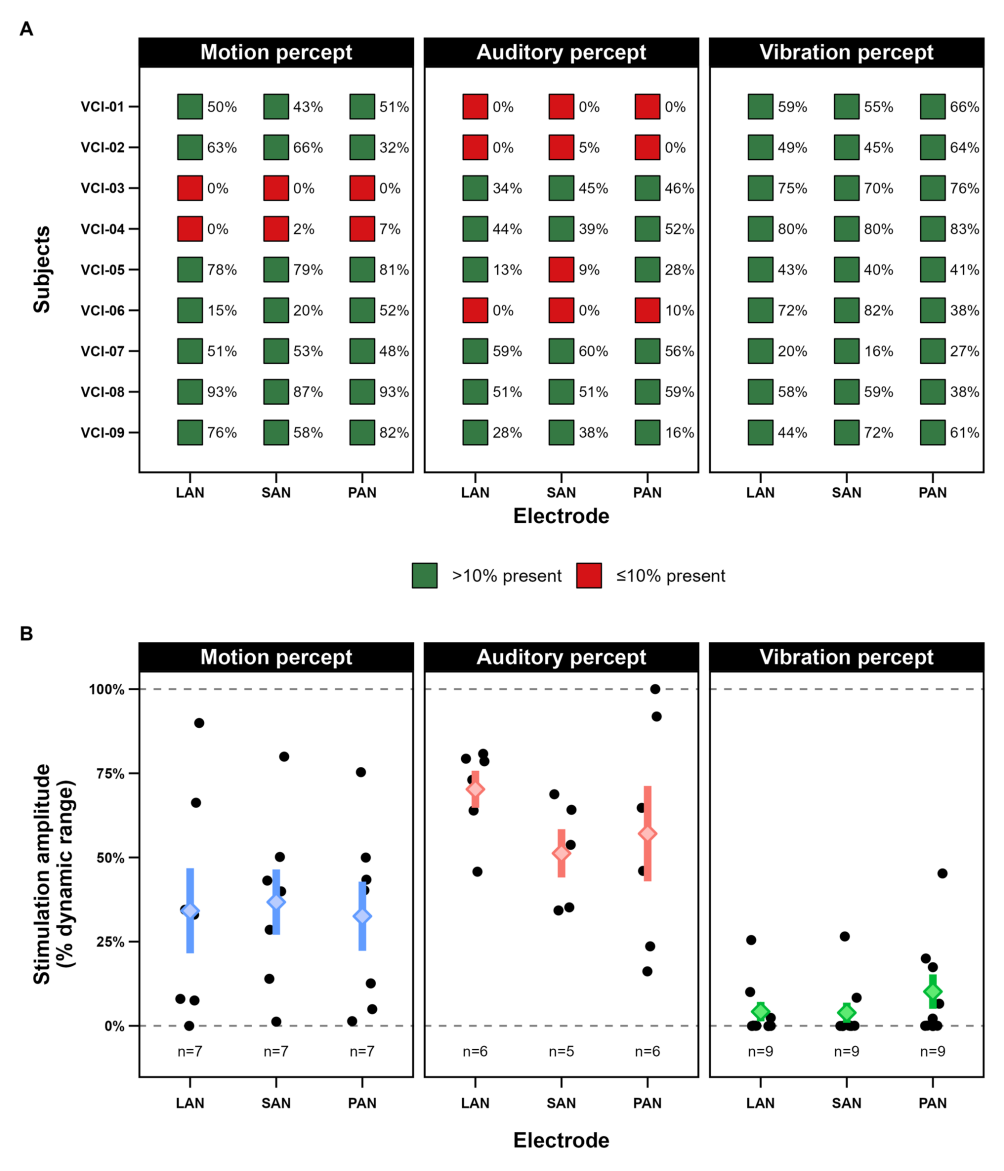
(**A**) Overview of the frequency of reported perceptions per subject (*y*-axis), per vestibular electrode (*x*-axis) and per perception type (motion, auditory, vibration). Green represents a perception type being significantly present (reported more than 10% across all given stimulations). Red represents a perception type being significantly absent (reported less or equal to 10% across all given stimulations), of the time across all given stimulations. (**B**) Overview of the individual and average perception thresholds of each perception type within the dynamic range. The colored bars with diamonds represent the mean threshold with whiskers delineating the standard error. Individual data points are plotted in black. VCI = vestibulocochlear implant, LAN = lateral ampullary nerve electrode, SAN = superior ampullary nerve electrode, PAN = posterior ampullary nerve electrode

Figure 1A presents an overview of these perception types and whether a perception type was significantly present (> 10% of presentations) per subject and per electrode. The frequency of the reported perception type is displayed as a percentage of the total number of given stimulations within a subject per vestibular electrode. Motion was perceived in seven out of nine subjects on all vestibular electrodes. In five out of nine subjects, an auditory percept was reported on all electrodes. One subject (VCI-05) significantly reported an auditory percept only on 2 out of 3 electrodes. All subjects perceived vibration on every electrode.

For subjects who experienced a given perception type, Fig. 1B illustrates both individual and average perception thresholds per type and per electrode, represented as a percentage of the dynamic range. On average, vibration perception occurs at the lower limit of the dynamic range and is often the first percept reported. Motion perception typically follows and generally emerges within the lower half of the dynamic range. In contrast, auditory perception tends to arise at higher stimulation levels, usually within the upper half of the dynamic range.

### Motion perception

**Fig. 2:**
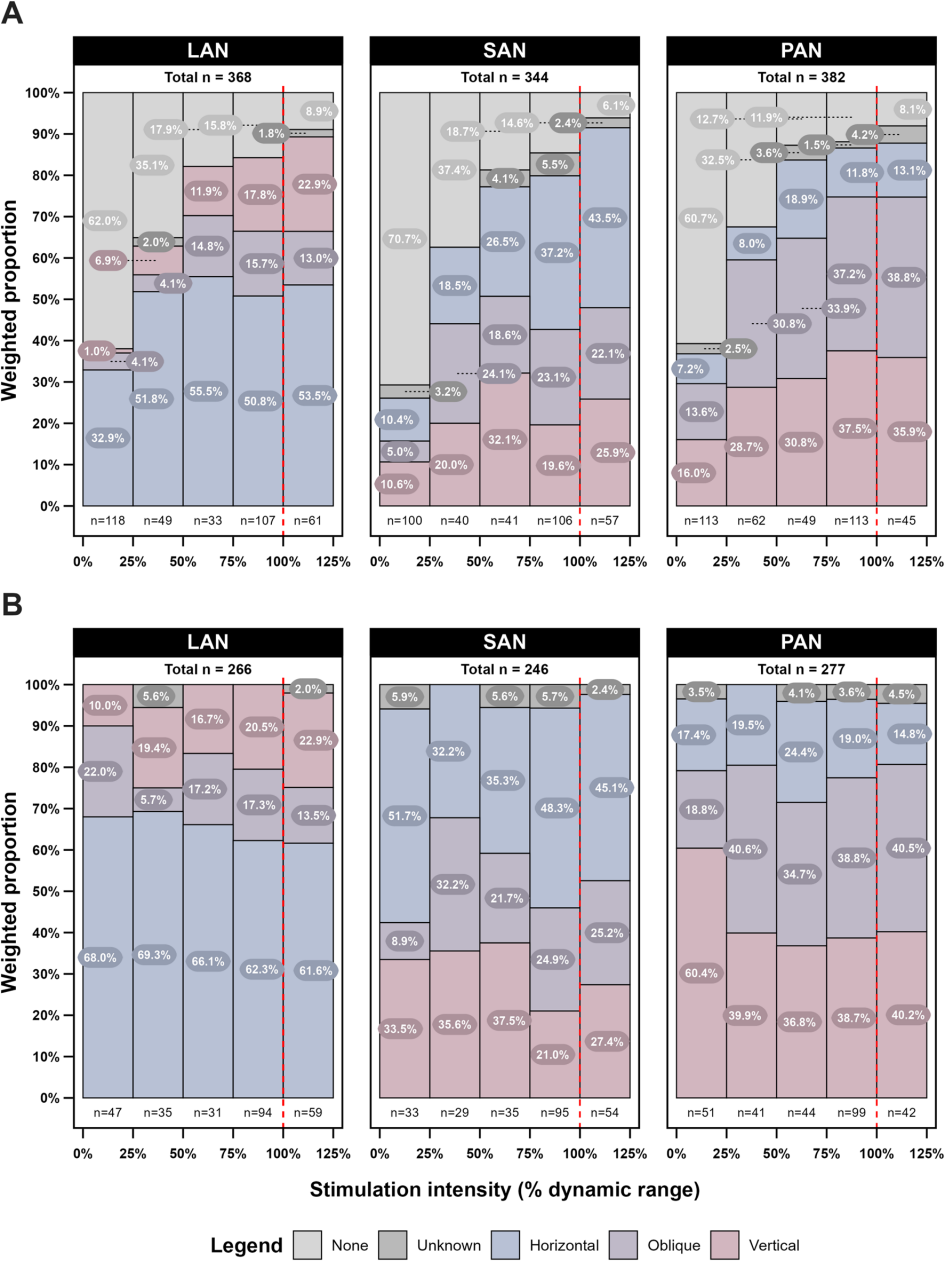
Weighted proportions (%) of reported movement direction categories per vestibular electrode across the dynamic range for all subjects combined. (**A**) Proportions including the ‘none’ category, and (**B**) proportions excluding the ‘none’ category after reweighting the data. Only subjects who reported motion perception were included (*n* = 7). The *x*-axis represents the stimulation intensity as percentage of the dynamic range over which stimulations were administered, where 0% corresponds to the vestibular threshold and 100% corresponds to the upper comfort level (red vertical striped line). The *y*-axis displays the weighted proportions of different movement direction categories reported by the subjects. LAN = lateral ampullary nerve electrode, SAN = superior ampullary nerve electrode, PAN = posterior ampullary nerve electrode

The weighted proportion of motion perception over the dynamic range per vestibular electrode is illustrated in Fig. 2**.** Only subjects who reported motion perception (*n* = 7) were included in this analysis. Motion perception increased with increasing stimulation intensity for all three vestibular electrodes (Fig. 2A). As described in the Methods section, three distinct main categories were identified for the quality of movement percepts, all based on the direction of movement: horizontal, oblique, and vertical. LAN stimulation resulted mainly in the perception of a horizontal movement, while SAN and PAN stimulation were generally reported as vertical and oblique movements. For example, in two subjects, VCI-08 and VCI-09, a Dix-Hallpike-like sensation was noted during PAN stimulation, demonstrating alignment of the reported movement percept with the stimulated canal. Only two subjects, VCI-01 and VCI-06, reported dizziness during some stimulations. However, this percept was not reported consistently across all test sessions by either subject.

To assess changes in perceived movement direction across the dynamic range, proportions were recalculated after excluding all stimulations in which no motion perception was reported (‘none’ category, see Fig. 2B). When LAN was stimulated and motion perception was present, a horizontal movement was perceived in 78.4% of stimulations at the lower end of the dynamic range (0–25%). This decreased to 69.1% at 25–50% of the dynamic range and 65.2% at the higher end of the dynamic range (75–100%). Conversely, the perception of an oblique and/or vertical movement increased from 21.6% at the lower end of the dynamic range (0–25%), to 34.8% at the higher end of the dynamic range (75–100%).

For SAN stimulation, the inverse of LAN stimulation was found with increasing stimulation amplitude. In case motion perception was present, 46.7% vertical/oblique movements and 43.7% horizontal movements were reported when stimulating between 0 and 25% of the dynamic range. At 25–50% of the dynamic range, vertical/oblique movement perception rose to 68.8% and horizontal movement perception decreased to 31.3%. Stimulating in the higher end of the dynamic range (75–100%) resulted in a decreased perception of 41.1% vertical/oblique movements and 53.8% horizontal movements.

No specific change or trend in weighted proportions between vertical/oblique or horizontal movements was found when stimulating PAN with increasing stimulation intensity.

### Auditory perception

**Fig. 3:**
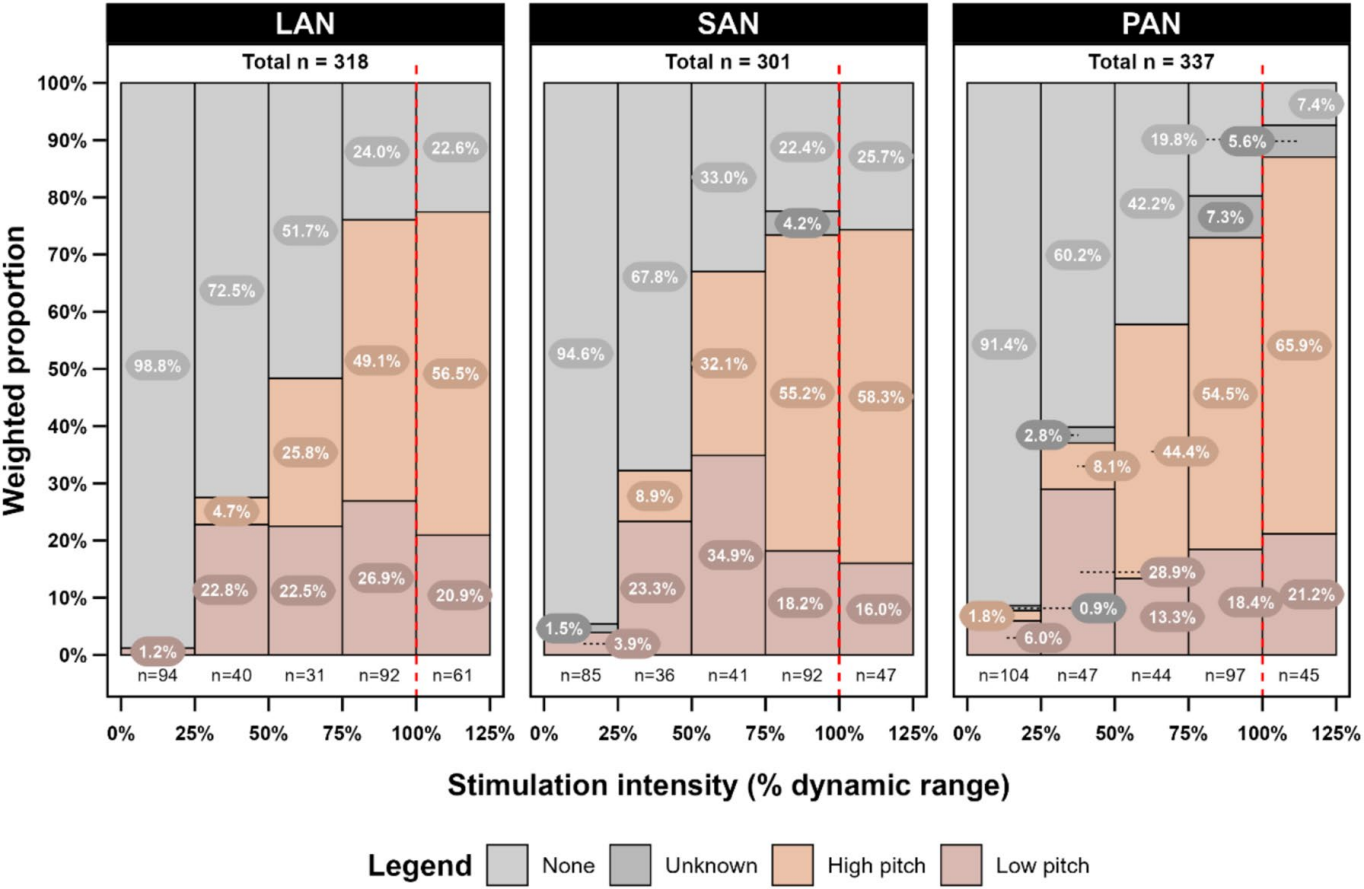
Weighted proportions (%) of auditory perception categories per vestibular electrode across the dynamic range for all subjects combined. Only subjects who reported auditory perception were included (*n* = 6). The *x*-axis represents the percentage of the dynamic range over which stimulations were administered, where 0% corresponds to the vestibular threshold and 100% corresponds to the upper comfort level (red vertical striped line). The y-axis displays the weighted proportions of different auditory perception categories reported by the subjects. LAN = lateral ampullary nerve electrode, SAN = superior ampullary nerve electrode, PAN = posterior ampullary nerve electrode

The weighted proportion of auditory percept quality on a group level per vestibular electrode is presented in Fig. 3. Only subjects who reported auditory perception were included (*n* = 6). Auditory perception increased with increasing stimulation amplitude. As stated in the methods, two distinct quality categories within auditory perception could be identified: high-pitched and low-pitched sounds. Across all vestibular electrodes, the occurrence of a high-pitched sound percept increased with higher stimulation amplitudes.

### Vibration perception

**Fig. 4:**
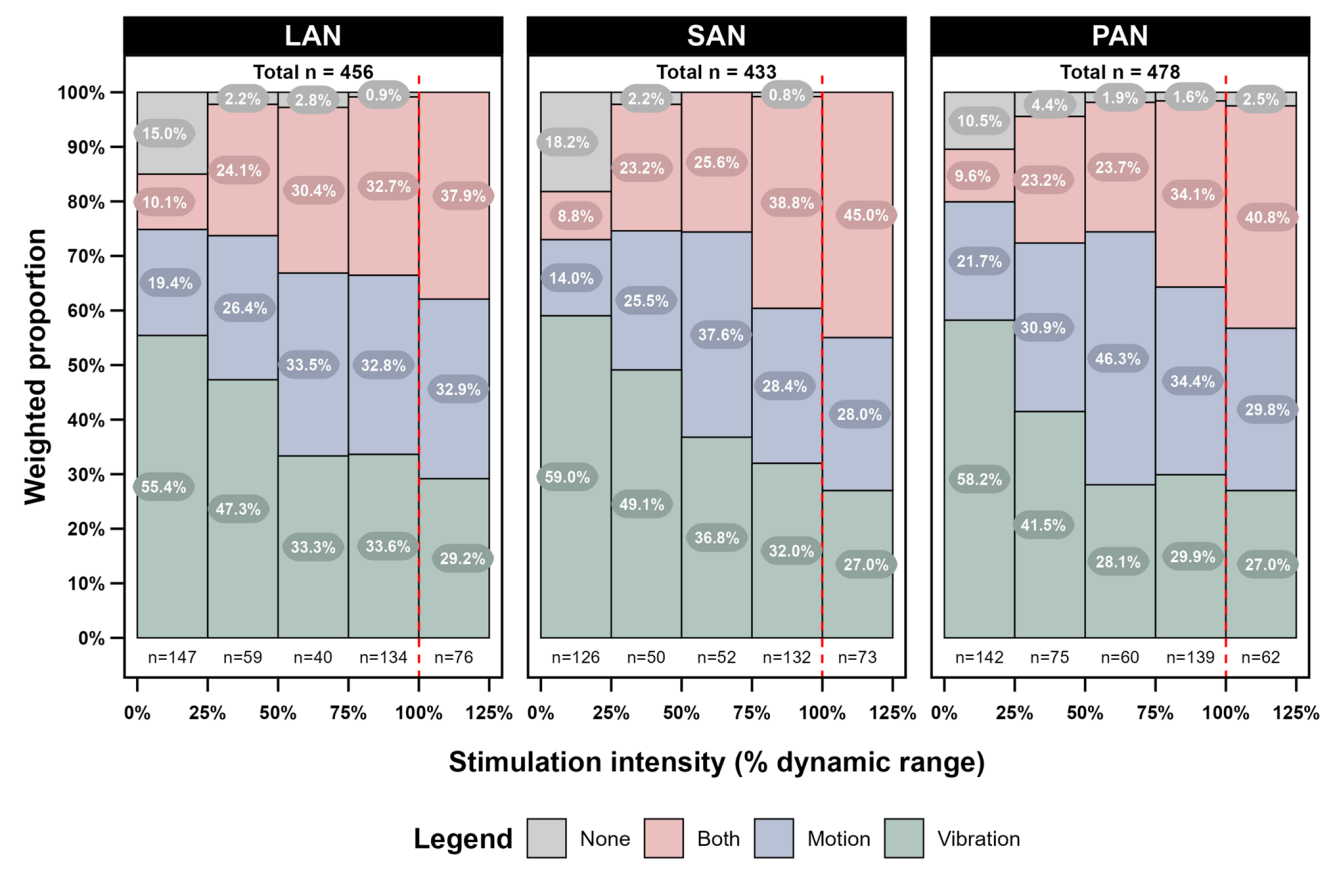
Weighted proportions (%) of perception of vibration, motion, or both vibration and motion per vestibular electrode. The x-axis represents the percentage of the dynamic range over which stimulations were administered, where 0% corresponds to the vestibular threshold and 100% corresponds to the upper comfortable limit (red vertical striped line). The y-axis displays the weighted proportions of different types of perception reported by the subjects. LAN = lateral ampullary nerve electrode, SAN = superior ampullary nerve electrode, PAN = posterior ampullary nerve electrode

The weighted proportions of vibration perception in relation to the perception of motion at the group level are presented in Fig. 4. As the stimulation amplitude increased, the vibration percept alone decreased across all vestibular electrodes. Specifically, the perception of vibration alone decreased from 55.4% to 33.6% at LAN, 59.0% to 32.0% at SAN, and 58.2% to 29.9% at PAN. Concurrently, the perception of motion only increased, rising from 19.4% to 32.8% at LAN, 14.0% to 28.4% at SAN, and 21.7% to 34.4% at PAN. Additionally, the perception of vibration combined with motion ("both" in Fig. 4) showed an increase, progressing from 10.1% to 32.7% at LAN, 8.8% to 38.8% at SAN, and 9.6% to 34.1% at PAN, as the stimulation intensity shifted from lower (0–25% of the dynamic range) to higher levels (75–100% of the dynamic range).

### Perception intensity

**Fig. 5:**
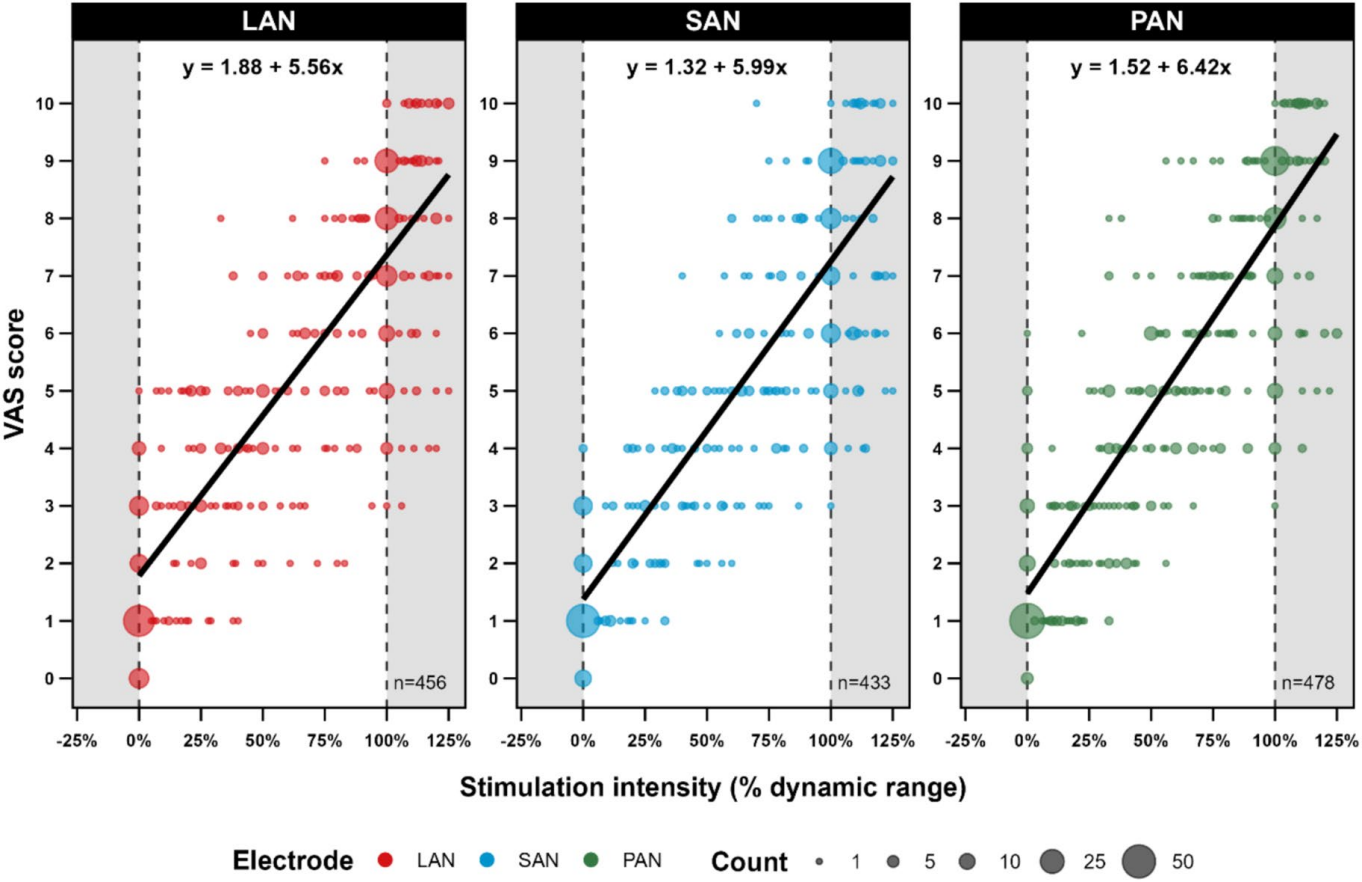
Perception intensity as a function of the stimulation amplitude (% of DR) for each vestibular electrode. The *x*-axis represents the percentage of the electrical dynamic range: 0% equals vestibular threshold, 100% equals upper comfortable limit. The *y*-axis represents the Visual Analog Scale (VAS) scores for perception intensity: zero represents no perception, 10 equals uncomfortable perception. Each dot corresponds to an individual observation, with the size of the dots reflecting the frequency of observations at that point. Linear regression lines for each electrode are shown in black. The formula for each linear regression is printed at the top of the plot. LAN = lateral ampullary nerve electrode, SAN = superior ampullary nerve electrode, PAN = posterior ampullary nerve electrode

Results regarding the intensity of the reported perception, scored on a VAS ranging from 0 (no percept) to 10 (uncomfortable) for each given stimulation is illustrated in Fig. 5. At UCL, the mean VAS score was 7.13 (range: 4–9) for LAN, 7.11 (range: 3–9) for SAN, and 7.64 (range: 3–9) for PAN stimulation.

A mixed linear regression model was fitted to predict VAS scores by stimulation intensity for each electrode. The model included a random intercept per subject and study visit as a random effect to account for individual variability. The intercepts, corresponding to a stimulation intensity of 0% (percentage of the dynamic range), were estimated as follows: 1.88 (95% CI: 1.33–2.42, *p* < 0.001) for the LAN electrode, 1.32 (95% CI: 0.77–1.86, *p* < 0.001) for the SAN electrode, and 1.52 (95% CI: 0.32–2.83, *p* < 0.001) for the PAN electrode. The regression slopes, indicating the average change in VAS scores for each additional percentage of stimulation intensity, were estimated as 5.56 (95% CI: 4.67–6.44, *p* < 0.001) for the LAN electrode, 5.99 (95% CI: 5.10–6.87, *p* < 0.001) for the SAN electrode, and 6.42 (95% CI: 5.54–7.30, *p* < 0.001) for the PAN electrode. The intraclass correlation coefficient (ICC) for the model was 0.96, indicating that 96% of the variance in VAS scores was due to differences between subjects, highlighting the importance of accounting for subject-specific variability in the analysis.

## Discussion

This study structurally characterized the perceptions reported by subjects in response to short pulses of electrical vestibular stimulation. It was found that three main types of perception were elicited: motion, auditory, and vibration. Motion perception was further categorized into horizontal, oblique, and vertical movements. Stimulation of LAN primarily resulted in the perception of horizontal movements, while stimulation of SAN and PAN mainly elicited perception of vertical and oblique movements. Furthermore, the intensity of motion, auditory, and vibration perceptions increased with higher stimulation amplitudes. Auditory perception typically began as a low-pitched sound, transitioning to a high-pitched sound as the stimulation increased across the dynamic range. In contrast, vibration perception decreased with increasing stimulation amplitude.

### Motion perception

Almost all subjects reported motion perception in response to electrical vestibular stimulation. These perceptions were generally well aligned with the approximate orientation of the stimulated semicircular canal. For LAN stimulation, the majority of reported movement directions were horizontal. For SAN and PAN stimulation, most directions were either vertical or oblique.

However, not all perceptions were perfectly aligned with the stimulated semicircular canal. At 0–25% of the dynamic range, there was greater misalignment compared to 25–50% of the dynamic range, suggesting that the stimulation intensity may have been insufficient to generate a clear movement direction. This effect was most pronounced in SAN stimulation and less evident in LAN and PAN stimulations (Fig. 2B). As the stimulation intensity increased to 25–50% of the dynamic range, misalignment of perception decreased, but rose again at the higher end of the dynamic range (75–100%).

At the higher end of the dynamic range, LAN stimulation resulted in increasing proportions of vertical and oblique sensations. Conversely, SAN stimulation resulted in a growing proportion of horizontal sensations. For PAN, a minority of motion percepts were horizontal, indicating some misalignment. Unlike LAN/SAN, this proportion remained relatively stable across intensity bins. This perception misalignment at the higher end of the dynamic range is most likely due to current spread. Current spread is a common issue in electrical stimulation of the nervous system and leads to activation of non-target nerve branches at high current levels. For vestibular implant stimulation, this can result in activation of the facial nerve, cochlear nerve, or other ampullary nerves. Anatomically, the LAN and SAN electrodes are in close approximation to each other. This anatomical relationship could facilitate current spread from one semicircular canal to the other, leading to perception misalignment.

To date, the effects of current spread in vestibular stimulation were primarily described in relation to VOR misalignment [40, 41, 47–49]. This study also indicated that current spread affects perceived perceptions, causing misalignment. Various strategies to minimize current spread were previously proposed, including optimizing electrode positioning, pre-compensation, and reducing current [48, 50]. Further research is needed to determine if perception misalignment is related to VOR misalignment and if the proposed strategies to reduce current spread also help minimize perception misalignment.

Apart from current spread, another factor contributing to the misalignment of perception could be the experimental setup. In the current setup, subjects were tested while sitting upright (without moving) with video-oculography goggles. This setup isolated the vestibular input by eliminating other sensory input, such as visual or proprioceptive information. This isolation was useful to study perceptual responses selectively and without interference, but might have also introduced a mismatch between these sensory inputs (i.e., vestibular system senses movement, while the somatosensory system senses no movement). As stated before, (self)-motion perception does not arise from the vestibular system alone but is integrated with other sensory input on multiple levels. A mismatch between these sensory inputs might also cause perception misalignment, reduced motion perception, or other discomforts such as dizziness. In real-life scenarios where subjects will be actually moving during stimulation, multisensory information (provided by other senses) might help enhance sensory perception in the correct plane of motion and diminish adverse effects of current spread.

Misalignment could also arise from imprecision in reported perceptions. Since this was the first comprehensive study into electrically elicited percepts, an open approach was used during subject interviews. This approach allowed for unbiased responses, but could also lead to missing information, especially when motion perception was subtle or barely noticeable. In such cases, subjects often found it challenging to describe their perceptions accurately, leading to potential inaccuracies. It was already established that subjects have difficulties describing vestibular symptoms in general, which could add to further inaccuracies [51].

In addition to the reporting challenges, the open approach complicated the differentiation between specific types of movements during analysis, such as distinguishing rotations from translations or tilt. For instance, the distinction between a horizontal translation and horizontal rotation was not always adequately reported, potentially leading to inaccuracies. This difficulty in categorization is why the study focused on broad categories like horizontal, vertical, and oblique movements as outcome measures, rather than attempting to classify more specific subcategories.

Another drawback of the open approach is that specific percepts such as vibration, motion, and auditory experiences might have been present but unreported due to the lack of targeted questioning. While this issue appears to be less significant for motion and auditory perceptions, it seems more problematic for vibration reporting, potentially leading to inaccuracies. Because of this, an overlap category between vibration and motion perception was made in Fig. 4.

Considering the limitations stated above, a more structured approach to interviewing subjects about their perceptions is recommended. Based on the findings of this study, a more closed set of categories could be adopted. The categorization structure from Table 2 can serve as a starting point for this structured approach. Furthermore, a separate VAS score should be obtained for each reported category: motion, auditory, and vibration. Subjects might also be trained in reporting these perceptions. This will most likely improve reporting accuracy, although some nuances might be lost.

Two subjects, VCI-03 and VCI-04, did not report a significant amount of motion perception. No definitive reason was identified. One possible explanation could be electrode positioning, but the vestibular electrodes were positioned at the desired location in all cases [46]. Another possible explanation could be the etiology of BV and duration of BV symptoms. Both subjects were diagnosed with DFNA9, a hereditary form of progressive sensorineural hearing loss associated with vestibular dysfunction. It is caused by pathogenic variants in the COCH gene, which encodes the protein cochlin. DFNA9 leads to significant degeneration of sensory hair cells and Scarpa ganglion cells, which are essential for signal transmission [52]. Additionally, eosinophilic deposits and structural atrophy in the vestibular system disrupt normal cellular architecture [52]. The extensive damage caused by DFNA9 could impair the effectiveness of electrical stimulation, possibly explaining the lack of significant motion perception in these subjects, despite proper electrode positioning. However, further research in larger cohorts is necessary to confirm this etiology-related hypothesis, since not all subjects with DFNA9 in this study showed a lack of motion perception. Additionally, the disease duration might play an important role, as prolonged disease duration could lead to reduced motion perception due to the progression of the disease. Notably, subjects VCI-03 and VCI-04 have disease durations of 30 and 10 years, respectively, which are the longest durations observed among the DFNA9 cohort in this study.

### Auditory perception

Auditory perception was reported by two-thirds of the subjects and typically started as a low-pitched sound that transitioned to a high-pitched sound as stimulation progressed over the dynamic range. The high-pitched sounds perceived at higher stimulation intensities could be attributed to the anatomical positioning of the electrodes and the tonotopy of the cochlea. The vestibular electrodes, implanted in the ampullae of the semicircular canals, are located close to the cochlea’s base. As described, higher stimulation amplitudes caused more current spread, potentially activating auditory hair cells in the base of the cochlea due to its proximity. Since the base of the cochlea is sensitive to high-frequency sounds, this could explain the high percentage of reported high-pitched sounds at the upper end of the dynamic range. Furthermore, distinguishing between vibration perception and a low-pitched sound was often challenging for subjects. During analysis, differentiating between a vibration or auditory perception posed a challenge when subjects reported a low-pitched sound such as a hum. This overlap might lead to over- or underestimations of the reported low-pitched sound and vibration perceptions, complicating the accurate characterization of these two perceptions. Especially at the lower end of the dynamic range, overlap between a low-pitched auditory perception and vibration perception might exist, as both low-pitched and vibration perceptions decrease over the dynamic range. This may confirm that these perceptions at low intensities are not accurate and become clearer at higher intensities, where they can be more accurately characterized.

Interestingly, the findings observed here do not completely align with findings reported in previous literature. Phillips et al. [42] observed that subjects did not experience auditory sensations during acute, semicircular canal stimulation alone. However, during combined vestibular and cochlear stimulation, vestibular stimulation influenced the perceived pitch and loudness during cochlear stimulation, indicating some crosstalk. Moreover, de Miguel et al. [53] concluded that there is no significant current flow from the cochlea to the vestibule or vice versa. They also reported that no subjects encountered the perception of sound during vestibular-only stimulation. It should, however, be noted that in this last study, lower stimulation intensities and stimulation rates were used. Furthermore, electrodes were placed in the vestibule near the otolith organs, while the current study has electrodes placed in the semicircular canals.

However, our own findings suggest that excitation spread between the cochlea and semicircular canals can occur at stimulation levels within the clinical fitting range for both vestibular and cochlear electrodes, as demonstrated by electrically evoked compound action potential (eCAP) measurements in vestibulo-cochlear configurations. Notably, the presence of eCAPs did not correlate with either misalignment of the electrically evoked VOR or the occurrence of auditory percepts during vestibular stimulation [54]. Although it was not always asked specifically, many subjects reported an increase in auditory perception intensity with increasing stimulation amplitude. This suggests that continuous auditory disturbances are unlikely during typical device use, as high stimulation usually occurs only during very quick head movements, depending on the fitting. However, if auditory perceptions cause too much discomfort, this might be a reason to adapt the fitting of the device. For instance, VCI-09 experienced discomfort, reporting a VAS score of 10 due to auditory perception, limiting the dynamic range. While intense auditory perceptions at high stimulation levels could potentially cause discomfort, these perceptions are infrequent and transient, potentially allowing for adaptation over time. These characteristics are favorable and enhance the potential for long-term use of the device without causing significant discomfort. To further investigate this, it is recommended that future research include a separate VAS for auditory perception, as stated above. Additionally, the subcategories of sound quality should be better defined, as current categorization is limited to low and high pitch. Moreover, the effects of auditory perception should be examined, particularly in the context of long-term use.

### Vibration perception

All subjects perceived some form of ‘vibration’ sensation upon stimulation, as illustrated in Fig. 1. This vibration was often one of the first perceptions reported, starting at threshold and persisting throughout the dynamic range. Subjects often found it challenging to describe this unfamiliar sensation, leading to varied descriptions such as vibration, current, buzz, wave, and hum. It is believed that these different descriptions refer to a common underlying sensation.

During analysis, it was sometimes challenging to determine if vibration perception persisted at higher levels of the dynamic range. At these levels, strong movement or auditory perceptions may have masked the vibration sensation. Furthermore, subjects occasionally displayed inattention during stimulation, leading to uncertainty in their reports about the presence of vibration when asked. Moreover, the perception of vibration was not systematically asked, which could contribute to inconsistencies in reporting. Finally, differentiating between a vibration or auditory perception when the subjects reported a low hum was often challenging, as stated above. Consequently, the analysis of vibration was not always reliable, sometimes necessitating extrapolation. Nonetheless, the decrease in reports of vibration over the dynamic range implies that the sensation may become less noticeable with prolonged exposure, further suggesting the device’s suitability for extended use without causing significant discomfort.

### Intensity

For all subjects, perception intensity started low and gradually rose higher towards UCL. This increase was linear and almost equal between all vestibular electrodes. This finding validates the feasibility of using a linear, stepwise approach to determine the dynamic range. However, significant variability was present in the VAS scores at UCL level among subjects and between electrodes. Regarding interindividual differences: this variability arises from whether the UCL was determined by VAS or by reaching the facial nerve stimulation threshold. Additionally, these differences likely result from variations in central processing of perceptions among individuals. Regarding differences between electrodes: perception intensity at threshold was significantly lower for SAN and PAN, compared to LAN. However, these differences were very small and are unlikely to be clinically relevant. Further analysis of variability in perceptions was beyond the scope of this article.

### Limitations and future prospects

While several limitations were already addressed above, this study did not consider temporal variability. Since responses were collected on multiple occasions, it is possible that these responses might vary over time, as well as due to more frequent use and increased experience with vestibular stimulation. Furthermore, intersubject variability was not explored. Both aspects were beyond the scope of this study. Nevertheless, these factors should be investigated in follow-up studies and with larger cohorts to gain a more comprehensive understanding.

Perception already plays an important role in determining the dynamic range during VI fitting, and this article further emphasizes its importance. While perception is already used to establish dynamic range, it can also be instrumental in optimizing other stimulation parameters. An important consideration for future investigations is the influence of varying stimulation parameters on perception. Systematically altering parameters such as pulse width, frequency, and amplitude could provide deeper insights on their effect on perception. These insights might help further optimization of VI settings for individual subjects. Currently, the VOR is mostly used to fit the VI and determine transfer functions and stimulation parameters for vestibular stimulation. It was previously hypothesized that relying on the VOR alone may be inadequate [40]. For example, if perceptual responses were used as a dependent measure instead of VOR, resulting transfer functions and stimulation parameters might differ significantly. As more experience is gained with perception in both acute and continuous electrical vestibular stimulation, it may be valuable to explore how these perceptual responses can be further integrated into the VI fitting process.

As mentioned, subjects often find it hard to describe (vestibular) perception, both in general and when being electrically stimulated. Future research should therefore aim to develop more structured methods to capture these perceptions and train subjects in reporting perception. Implementing the recommendations described above is likely to enhance the accuracy and reliability of the collected data.

## Conclusion

This study systematically characterized perceptual responses to electrical vestibular stimulation in VCI subjects for the first time. Three main types of perceptions were identified: motion, auditory, and vibration. Motion perception generally aligned with the stimulated semicircular canal, though increasing misalignment occurred at higher amplitudes, likely due to current spread. Auditory perceptions transitioned from low to high pitches at higher stimulation levels, indicating possible crosstalk with cochlear pathways, while overlap with vibration perception may occur at lower stimulation levels. Vibration was consistently reported by all subjects, mainly around threshold levels.

These findings demonstrate that perceptual responses can be systematically evaluated and provide valuable insights into the effects of vestibular electrical stimulation. They also demonstrate that perception might be as significant an outcome measure as the VOR for the fitting of vestibular implants. Future studies should explore how different stimulation parameters influence perception, as understanding these relationships is likely to support the optimization of VI settings for individual subjects. Additionally, employing structured methods for interviewing, such as using the proposed perception categories, and training subjects in accurately reporting their perceptions, could significantly improve data quality and contribute to more effective VI fitting processes.

## Data Availability

The raw data supporting the conclusions of this article are available from the corresponding author upon reasonable request.
